# Elevated romantic love and jealousy if relationship status is declared on Facebook

**DOI:** 10.3389/fpsyg.2015.00214

**Published:** 2015-02-26

**Authors:** Gábor Orosz, Ádám Szekeres, Zoltán G. Kiss, Péter Farkas, Christine Roland-Lévy

**Affiliations:** ^1^Department of Social and Developmental Psychology, Institute of Psychology, Eötvös Loránd UniversityBudapest, Hungary; ^2^Institute of Cognitive Neuroscience and Psychology, Research Centre for Natural Sciences, Hungarian Academy of SciencesBudapest, Hungary; ^3^Institute of Psychology, University of SzegedSzeged, Hungary; ^4^Département de Psychologie, Université de Reims Champagne-ArdenneReims, France

**Keywords:** facebook, romantic love, jealousy, relationship status, social network

## Abstract

Declared relationship status on Facebook can serve as a public commitment and as an extra layer of a couple’s security. However, the question arises: do those who report the relationship status feel stronger romantic love and jealousy toward their partners than those who do not share such information publicly? To test this assumption, profile information and questionnaire data of romantic love and jealousy were gathered from 292, 230 females) respondents that were in a relationship. Our results suggest that announcing the relationship status is associated with elevated romantic love and jealousy. Therefore, being “Facebook official” can be interpreted as a tie-sign indicating that the couple is “out of the market,” and can promote their unity as a “digital wedding ring.”

## INTRODUCTION

For heterosexual American couples who met in 2009, the Internet was one of three most common scenes of meeting. Whereas the proportion of couples whose first meeting was mediated by friends has decreased since the ’90s, the proportion of couples who met online has continuously increased during the last 20 years (Rosenfeld and Thomas, 2012). According to this representative study, 22% of the Americans reported that they first met online, which is approximately the same proportion of couples who met in bars. This ratio is more than two times larger than the number of couples who met in college. Besides the evolved custom of meeting someone online before making a more direct contact, other forms of internet-assisted relationship initiations are also relevant. For example, according to Sprecher (2009) young adults search for a person’s social network profile after the first unintentional oﬄine encounter. The results from Fox and Warber (2013) suggest that the sequence of internet-assisted romantic escalation starts with a face to face meeting continues with different online interactions and results in dating. However, in several cases, this sequence can be disturbed if the “target” has an “In Relationship” status.

Years ago, people did not walk around with a direct sign that stated their relationship status, except for the visibility of the wedding ring. However, they used so-called tie-signs (Goffman, 1971) that are acts, objects or expressions, which make evident the nature of a relationship (i.e., wedding ring, facial expressions, body proximity). Since ancient Egyptians, Greeks, and Romans the wedding ring has symbolized *“the highest form of trust”* (Chesser, 1980, p. 205)^1^. Today it represents the couple’s bonding and never ending love. This is the most popular tie-sign, which is an essential component of a wedding ceremony. This ceremony can be interpreted as the main public announcement of the partners’ commitment in front of a large audience. In modern online environments, such as Facebook, the relationship status may convey similar meanings, because in online settings it can also be interpreted as a tie-sign which *“typically serves as a relational indicator to the dyad using the behaviors and to their audience”* (Afifi and Johnson, 2005, p. 190). Among those who seek a partner, relationship status is the first and most relevant piece of information on Facebook profiles (Fox et al., 2013). We assume that setting the relationship status (or being “Facebook official”) is the result of a deliberated decision, similar to other personality-related information portrayed (Back et al., 2010). We also need to consider Papp et al.’s (2012) results which showed that posting relationship status is a major factor for women’s relationship satisfaction. Based on Fox et al.’s (2013) results, published relationship status can be interpreted as a social and interpersonal commitment, and as a sign that the couple is “off the market.” We assume that relationship status conveys information regarding the quality of the relationship in terms of love and jealousy.

Previous studies showed that individuals are motivated to keep in contact via Facebook with existing romantic partners; they can also be motivated by seeking potential future partners (Rau et al., 2008). Relatively few published studies examined the link between Facebook use and love (Rau et al., 2008; Young et al., 2009; Bowe, 2010; Papp et al., 2012; Fox et al., 2013) or jealousy (Muise et al., 2009; Marshall et al., 2012). One of the most important pieces of information that a Facebook profile can present regarding love is the public relationship status. According to Bowe’s (2010) study, when a couple appears in many photos together, it is more likely that the partner’s name will be displayed on their own profile. However, differences in romantic love and jealousy between those who provide relationship status information and those who do not share this information publicly have been so far scarcely investigated (Papp et al., 2012; Fox et al., 2013).

Rubin’s (1970) romantic love concept and scale was used in order to measure experienced companionate love between Facebook users. According to this study, romantic love can be defined as love between unmarried opposite-sex peers that could potentially lead to marriage. Rubin’s (1970) scale contains one factor that has three main theoretical components: attachment, caring, and intimacy. Compared to other conceptualizations of love, such as Sternberg’s (1986) triangular love theory or Hatfield et al.’s (2008) distinction between passionate and companionate love, Rubin’s (1970) Romantic Love Scale and concept aims to grasp companionate rather than passionate love (Acker and Davis, 1992; Fehr, 1994; Hatfield et al., 2008). The concept of Rubin’s (1970) romantic love is suitable, when taking into account Bowe’s (2010) results that found that Facebook users change their status to “in relationship with” if they feel that the relationship progressed to a more mature, stable and “official” level. The public declaration of a relationship on Facebook can be interpreted as the acknowledgment of a mutual commitment to friends, family, and acquaintances. According to Bowe’s (2010) interpretation, it serves as an extra security layer in terms of engagement. In line with these results, Papp et al. (2012) found that declared relationship status is linked to women’s relationship satisfaction. Furthermore, according to their respondents, declaring the relationship status publicly protects the couple from potential outsider admirers, whilst making it harder for people to initiate flirtatious attempts with other’s partner. These aspects led to the second focus of the present study, the phenomenon of jealousy.

Jealousy can be defined as a negative emotion resulting from one’s love being lost to a rival, or the threat of that possibility (Mathes and Severa, 1981). Previous Facebook studies found that there is a positive correlation between the amount of time that someone in a relationship spends on Facebook and Facebook-induced jealousy^2^. Furthermore, Marshall et al. (2012) found that attachment anxiety is positively linked to Facebook jealousy, while avoidance is negatively linked. Other studies found that people who show lower levels of trust tend to suffer higher Facebook jealousy (Muise et al., 2009). While these studies focus mainly on the negative aspects of jealousy, others have also shown that love and jealousy are positively associated. On one hand, Facebook users post-information about their romantic relationship to express their commitment, and on the other hand they follow their partner’s status updates, Facebook friends or wall posts in order to preserve the relationship. However, excessive monitoring may be associated with negative outcomes such as oﬄine and online relational intrusion (Elphinston and Noller, 2011). Furthermore, such activities can contribute in ameliorating the feelings of uncertainty and the avoidance of threats from potential rivals (Tokunaga, 2011).

As the strongest cue on Facebook indicating commitment with a partner is the announcement of being in a relationship, we set our goal to examine the link between romantic love and jealousy in connection to the declaration of a relationship status. Taking into account both love and jealousy-related Facebook studies, we assume that individuals who announce their relationship status (with or without a name) will report higher scores on (a) romantic love, and on (b) jealousy scales. Furthermore, we aim at measuring how well posting relationship status as “in a relationship” predicts self-reported love and jealousy independently from variables such as gender, length of relationship, and Facebook use Intensity.

Hypothesized control variables for love included: jealousy, length of relationship, intensity of Facebook use, gender, and age for the following reasons. Jealousy as a control variable can be taken into account on the basis of previous studies (Mathes and Severa, 1981), as it is significantly related to love. Length of relationship was taken into account, because as Fox et al. (2013) found, it takes weeks or even months to declare publicly on Facebook a relationship status. The duration of the relationship can be an important control variable if the effect of the relationship status on love is examined. Controlling Facebook Intensity scores can also be important by assuming that those who use Facebook more intensively probably pay more attention to relationship posts than those who use Facebook less intensively. Therefore, it is supposed that the Facebook Intensity should be controlled in order to separate the independent effect of relationship status on Love. Finally, gender and age can also be important variables. Considering Papp et al. (2012) and Fox et al. (2013), men and women evaluate the meaning and importance of posting relationship status differently; men may find it less important to post a relationship status or to be “Facebook official,” than females.

In the case of jealousy we chose intensity of Facebook use, gender, and love as control variables. On the basis of Muise et al. (2009) it is supposed that more intensive Facebook use is related to elevated jealousy. Furthermore, regarding Facebook Intensity, it is supposed that, for those who use Facebook more intensively, it might be more important to indicate their relationship status publicly. We found useful to also control gender. On the basis of previous studies (Mathes and Severa, 1981) with this scale, men experience more jealousy than women. Furthermore, it was important to include gender in this analysis because Fox et al. (2013) found that (1) women saw being “Facebook official” as a more important step in the relationship than men; (2) women, more than men, felt that being Facebook official means that the relationship is exclusive; and (3) women were more likely to believe that people notice when a couple goes as “Facebook Official.” Therefore, controlling gender can be useful in order to measure the independent impact of relationship status on jealousy. Finally, love was also entered as control variable, because previous results (Mathes and Severa, 1981) suggested that love and jealousy are positively linked.

## MATERIALS AND METHODS

### PARTICIPANTS

The total sample consisted of 532 Hungarian participants (380 female), aged between 16 and 69 years (*M* = 24.88, SD = 6.94). Respondents were recruited by posting an online survey link on Facebook. Among them, we chose a subsample (*N* = 292 (230 female) ranging between 16 and 69 years-old (*M* = 24.92, SD = 6.62) who, based on the online survey, were currently in a relationship. In order to assess relationship status, respondents of the online survey answered the following question: “Are you currently dating with a romantic partner and/or living in a relationship?” by choosing the following possibilities: “No” (*N* = 181, 34.7%), “Rather no” (*N* = 23, 4.3%), “Rather yes” (*N* = 31, 5.8%), “Yes” (*N* = 292, 55.1%). We only analyzed the data of the individuals who answered “Yes” to this question. In this subsample 66 (22.6%) respondents displayed nothing in their relationship status, 8 (2.7%) displayed “Single,” 179 (61.3%) displayed “In a relationship,” 12 (4.1%) displayed “Engaged,” and 27 respondents (9.2%) displayed “Married.” In the regression analysis, respondents belonging to the group are those who indicated a relationship status on Facebook, by announcing either “In a relationship” or “Engaged” or “Married.” There were no gender differences regarding proportions of shared relationship statuses. The average length of the respondents’ relationship was 37.83 months (SD = 48.19). Nine respondents (3.6%) had an elementary school degree, 13 (17.4%) had a vocational school degree, 150 (59.3%) had a high-school degree, and 81 (32%) had a higher education degree. Among them only seven participants’ partner has not got a Facebook profile.

### DATA GATHERING AND MEASURES

The dataset was collected by a Facebook application based on Kosinski and Stillwell’s myPersonality application (Kosinski and Rust, 2011; Kosinski and Stillwell, 2012). The Concerto platform was provided by the Psychometric Center of University of Cambridge, which allowed us to acquire Facebook user information such as relationship status. Furthermore, participants got a personalized feedback on the scales that they completed, in exchange for their time and effort. Before starting the questionnaire, participants received detailed information about the study and a list of personal data gathered from their Facebook profiles. Subsequently, participants read and approved the informed consent.

The scales used were translated, and back-translated by Náfrádi and Orosz (in preparation) following Beaton et al. (2000) protocol. The first scale is the Rubin’s (1970) *Romantic Love Scale* (Náfrádi and Orosz, in preparation). The Hungarian version has one factor and it contains eight items (α = 0.83; 1 = “not at all true/disagree completely”; 9 = “definitely true/agree completely”)^3^. While this scale was constructed for being factorically unitary, it covers three aspects of romantic love: (a) affiliative and dependent need, (b) predisposition to help, (c) exclusiveness and absorption. The second scale is the Hungarian version of Mathes and Severa’s (1981) *Interpersonal Jealousy Scale* includes 12 items. It contains three factors: jealousy toward ex-partner (I become sad if I see a picture about X and his/her ex-partner), exclusivity (i.e., I feel possessive toward X), and anticipated infidelity [I don’t believe that if X would flirt with someone from opposite sex (R)]. In the present study, the subscales were not separated. Instead, we used the aggregated scores of the three factors (α = 0.84; 1 = “absolutely false/disagree completely”; 6 = “absolutely true/agree completely”)^4^. The last scale is Facebook Intensity Scale (FBI; Ellison et al., 2007), which assesses self-reported data regarding the extent to which participants engage in Facebook activities. This measure was needed because posting relationship status can be more relevant to intensive Facebook users than less intensive ones. Therefore, differences on this aspect can have impact on the inclination to post-relationship status. The *FBI* was translated to Hungarian in the above-mentioned manner. This scale contains eight items (α = 0.78) with three different Likert-type scales (for details see Ellison et al., 2007). In sum, the scales used have good construct validity and they are reliable in terms of their internal consistency.

## RESULTS

First, independent *t*-tests were performed to examine differences between participants who are in relationship but who do not declare their relationship status on Facebook and those respondents who declared their relationship on Facebook. In line with our assumptions, results indicate significant differences between the two groups on Romantic Love Scale and Interpersonal Jealousy Scale (see **Table 1**).

**Table 1:** Independent samples tests.

	Relationship status available on facebook			No relationship status available on facebook			*t*-test for equality of means			
	*N*	Mean	SD	*N*	Mean	SD	*t*	df	Mean difference	Significant
Romantic Love Scale	218	61.36	8.29	74	57.21	11.55	-2.85	290	-4.14	0.005*
Interpersonal Jealousy Scale	216	44.20	10.31	73	39.95	10.73	-2.96	287	-4.25	0.003*

Later, a hierarchical multiple regression analysis was conducted to evaluate how well posting relationship status as “in a relationship” predicts the results on Rubin’s (1970) Romantic Love Scale. The predictors were separated into two distinct sets. Block 1 contained four variables: jealousy scores, gender (dummy variable, coded as 1 = male, 2 = female), length of relationship (in months), and Facebook intensity (aggregated score) that can have an impact on love independently from relationship status, while Block 2 contained relationship status (dummy variable, coded as 0 = nothing or “Single,” 1 = “in a relationship, “Engaged,” or “Married”). Gender was coded as a dummy variable (0 = male, 1 = female). Only those predictors were included in the analysis which significantly correlated with love scores.

The control predictors and the relationship status together relate significantly to love scores *R*^2^ = 0.11, adjusted *R*^2^ = 0.09, *F*(6,282) = 5.63, *p* < 0.001. Only Jealousy *t*(288) = 4.03, *p* < 0.001 scores of the control predictors accounted for a significant amount of love scores variation, R^2^_change_ = 0.08, *F*_change_(5,283) = 4.99, *p* < 0.001, whereas sharing one’s relationship status *t*(288) = 2.86, *p* = 0.005 accounted for a significant amount of variance of love scores R^2^_change_ = 0.03, *F*_change_(1,282) = 8.16, *p* = 0.005 (**Table 2**). After controlling for the effects of Facebook Intensity scores, jealousy made a small, but significant independent, contribution to the variance in love scores. The multiple regression results suggest that beyond the effect of jealousy, those individuals who declare their relationship on Facebook report a more elevated love than those who are in a relationship but do not display their togetherness on Facebook.

**Table 2:** Summary of hierarchical regression analysis for variables predicting love (*N* = 289).

	Model 1			Model 2		
Variable	B	SE B	β	B	SE B	β
Gender	0.53	1.35	0.02	0.90	1.33	0.04
Facebook intensity	-0.07	0.08	-0.05	-0.11	0.08	-0.08
Jealousy	0.21	0.05	-0.24**	0.18	0.05	0.21**
Length of relationship	-0.019	0.01	-0.10	-0.02	0.01	-0.10
Age	-0.13	0.10	-0.09	-0.11	0.10	-0.08
Relationship status				3.59	1.26	0.17**
*R*^2^	0.08			0.11		
*F* for change in *R^2^*	4.99**			8.16**		

**p < 0.01.

Another Hierarchical multiple regression analysis was conducted from relevant control variables and relationship status in order to predict scores on Interpersonal Jealousy Scale. In this analysis, Block 1 included gender as a dummy variable (dummy coded as 0 = male, 1 = female), Facebook intensity scores and scores on the Rubin’s (1970) Romantic Love Scale^5^, while Block 2 contained relationship status as a dummy variable (coded as 0 = nothing or “Single,” 1 = “in a relationship, “Engaged,” or “Married”). Only those predictors were included in the analysis which significantly correlated with jealousy scores.

These control variables were chosen because, on the basis of Muise et al. (2009) it is supposed that more intensive Facebook use is related to elevated jealousy. Furthermore, regarding Facebook Intensity, it is supposed that, for those who use Facebook more intensively, it might be more important to indicate their relationship status publicly. We found useful to also control gender. On the basis of previous studies (Mathes and Severa, 1981) with this scale, men experience more jealousy than women. Furthermore, it was important to include gender in this analysis because Fox et al. (2013) found that (1) women saw being “Facebook official” as a more important step in the relationship than men; (2) women, more than men, felt that being Facebook official means that the relationship is exclusive; and (3) women were more likely to believe that people notice when a couple goes as “Facebook Official.” Therefore, controlling gender can be useful in order to measure the independent impact of relationship status on jealousy. Finally, love was also controlled, because previous results (Mathes and Severa, 1981) suggested that love and jealousy are positively linked.

The regression equation was significant regarding jealousy, *R*^2^ = 0.11, adjusted *R*^2^ = 0.09, *F*(4,284) = 8.55, *p* < 0.001. Gender *t*(288) = 2.64, *p* = 0.009 and Love *t*(288) = 3.49, *p* = 0.001 as control predictors accounted for a significant amount of jealousy scores, whereas tendency was measured in the case of Facebook Intensity *t*(288) = 1.71, *p* = 0.09; R^2^_change_ = 0.09, *F*_change_(3,285) = 9.81, *p* < 0.001. However, declaring a relationship status *t*(288) = 2.10, *p* = 0.037 is also accounted for a significant amount variability of jealousy scores R^2^_change_ = 0.02 *F*_change_(1,284) = 4.41, *p* = 0.037 (**Table 3**) .

**Table 3:** Summary of hierarchical regression analysis for variables predicting jealousy (*N* = 289).

	Model 1			Model 2		
Variable	B	SE B	β	B	SE B	β
Gender	3.69	1.51	0.14*	4.00	1.50	0.15**
Facebook intensity	0.19	0.09	0.12*	0.16	0.09	0.10
Love	0.26	0.07	0.23**	0.23	0.07	0.20**
Relationship status				3.03	1.44	0.12*
*R*^2^	0.09			0.11		
*F* for change in *R^2^*	9.81**			8.55**		

*p < 0.05; **p < 0.01.

These results suggest that declaring the relationship status publicly offers a small additional, independent predictive power of Interpersonal Jealousy scores beyond that contributed by control predictors. Therefore, the multiple regression results suggest that women, intensive Facebook users, and individuals who experience elevated love are more jealous. In addition to these effects, those who report their relationship status experience more jealousy compared to those who are in a relationship but do not indicate it on Facebook^6^.

We also examined further interactive effects of Facebook intensity scores, gender, length of relationship, age, love, and jealousy (respectively) on love and jealousy scores but none obtained statistical significance (love model: *p* > 0.23; jealousy model *p* > 0.89).

## DISCUSSION

The aim of the present study was to test the hypotheses that individuals who disclose relationship-related information report more pronounced romantic love and jealousy toward their partner than those who do not share such information publicly. The results supported our hypotheses since significant differences were found regarding love and jealousy between those who posted their relationship status publicly on Facebook and those who did not. Furthermore, both jealousy and love were predicted by the declaration of the relationship status. However, in both cases the explained variability was relatively low. The results contribute to previous findings (Bowe, 2010) claiming that announcing relationship status reflects a new dimension of the couple’s relationship in which partners acknowledge their commitment publicly. This result is in line with Fox et al.’s (2013) qualitative results which shows that being “Facebook official” is interpreted as the couple being “out of market.” One of their respondents stated that becoming “Facebook official” is a kind of ring for the new generation. Another respondent referred to it as a newly developed, well-established level of the relationship. Furthermore, according to these respondents, if someone is Facebook official it means that it is also official in the real life. Therefore, similar to Back et al.’s (2010) results, Facebook profiles not only reflect on actual personality for observers but seem to convey reliable information about the romantic life of Facebook users as well. Namely, making such an elaborate decision as being Facebook official by posting relationship status publicly indicates an elevated romantic love in terms of Rubin’s (1970) dimensions comprising attachment, caring, and intimacy.

Previous studies suggested on the one hand, that Facebook usage in itself can increase jealousy in a relationship [see for example Facebook jealousy by Muise et al. (2009)]; on the other hand, Tokunaga (2011) found that expressing a couple’s unity on Facebook can provide more security and reduce threats from rivals. However, there is no information available concerning temporal dynamics of jealousy and Facebook usage and it is not known how the feelings of jealousy might change before and after providing relationship status on Facebook. Individuals who tend to be more jealous might disclose relationship status on Facebook and then require this declaration from the partner to make them feel less insecure. Possibly, after the disclosure, their jealousy levels might decrease. However, it is also possible that after the declaration, jealousy remains at the same level as before because such an announcement can be considered as a weak protection from potential rivals. Furthermore, based on Muise et al.’s (2009) results, the majority of Facebook users have previous romantic or sexual partners added as friends on the site and more than 90% of the respondents claimed that their partner has friends whom they do not know, which might contribute to the elevated/sustained levels of uncertainty and jealousy.

According to Mathes and Severa (1981), the positive link between romantic love and jealousy is reasonable in stable relationships (in the present sample, the average length is more than 3 years). Individuals who feel strong romantic love are more sensitive to threats to their relationship in different contexts. Facebook is such a special social context, which facilitates the interaction with the ex-partners and potential rivals. Due to these characteristics, Facebook itself can undermine the stability of romantic relationships. On one hand, those individuals who feel intense romantic love toward their partner are more jealous in this context; on the other hand, for these very same reasons, they are more motivated to express their commitment on Facebook, in order to protect the relationship.

Even if there might be more relevant potential control variables beyond the ones used, it can be valuable to mention the effect of the control predictors of the regression models. Hatfield et al.’s (2008) study showed that romantic love had eroded by 1 year, whereas Sprecher (1999) found that in a 5-years term romantic partners globally perceive that their love, commitment, and satisfaction increased. However, such an increase was not supported by Sprecher’s (1999) measures which we used here. Our cross sectional data suggest the lack of relationship. Further investigation might be needed with alternative scales which can separate the effects of different dimensions of love. Regarding gender differences, results suggest that jealousy is higher among women than men, which is in line with Muise et al.’s (2009) findings on Facebook jealousy.

Finally, on the basis of the present results we have no information on causality: it is unclear whether stronger love and/or jealousy predicts the declaration of relationship status on Facebook, or whether the announcement of the relationship status deepens love and makes women more jealous. Is love impacted by the announcement of the relationship through jealousy or is it the opposite? Beyond the lack of causality, the present study has several limitations due to the unrepresentative sampling and the self-reported data about feelings (love, jealousy). Other limitations are lack of information about their partners and small effect sizes. Furthermore, it would be interesting to see quantitatively in what form expectations from the partner and norms of peer groups have an impact on declaring relationship status on Facebook. Considering the shortcomings of the present study, future research should examine these questions with alternative measures, such as multidimensional love scales (e.g., Sternberg Triangular Love Scale) and Muise et al.’s (2009) Facebook Jealousy Scale. The Sternberg Love Scale might be more appropriate instead of Rubin’s (1970) scale because it can distinguish three dimensions of love, in terms of passion, intimacy, and commitment. It would be interesting to see which dimension is more affected by the relationship status. Muise et al.’s (2009) Facebook Jealousy Scale could provide a context-specific measurement regarding the detailed activities that could potentially invoke jealousy in the often ambiguous public Facebook environment. Finally, individual differences in terms of extraversion can be related to posting relationship status. Further studies are needed to clarify the role of individual differences in posting relationship status.

In summary, those who declare their relationship on Facebook—with or without the name of the partner—report stronger romantic love toward their partner than those who are not “Facebook official.” In addition to the stronger love, they also report higher levels of jealousy, which can indicate their intentions to protect the relationship. We conclude that disclosing relationship status is a modern, online tie-sign. It can be interpreted as an expression of commitment which reflects on a new, more stable phase of the relationship with stronger romantic love and jealousy. Therefore, being a Facebook official can be interpreted as a “digital wedding ring” or one of the virtual maturity indicators of the advancement in a relationship.

## Conflict of Interest Statement

The authors declare that the research was conducted in the absence of any commercial or financial relationships that could be construed as a potential conflict of interest.

## Footnotes

1 *Nota bene:* a possible reason for wearing the ring on the fourth finger, named the “Ring finger,” is an old belief that a small vein (vena amoris) runs straight to the heart from the fourth finger, making it the most appropriate place to wear the pledge of love (Chesser, 1980). Furthermore, according to Lacey (1969) the word “wedding” derives from “wed” which means pledge.

2 Facebook jealousy is the experience of jealousy in the specific context of Facebook and often results in the excessive surveillance of the partner’s profile (Muise et al., 2009).

3 In confirmatory factor analysis of the Hungarian version (*N* = 599) of the Romantic Love Scale has appropriate model fit indices (χ^2^/df = 2,583; RMSEA = 0.053; CFI = 0.983; TLI = 0.969) and internal consistency (α = 0.88).

4 In confirmatory factor analysis of the Hungarian version (*N* = 599) of the Interpersonal Jealousy Scale has appropriate model fit indices (χ^2^/df = 2,157; RMSEA = 0,044; CFI = 0.976; TLI = 0.965) and internal consistency (α = 0.78).

5 Age and length of relationship was unrelated to jealousy scores.

6 Neither love, nor jealousy results are affected by inclusion or exclusion of those respondents who reported in the questionnaire that they are “rather in a relationship” and with the inclusion or exclusion of those who indicated “married” or “engaged” as Facebook relationship status.
